# Effects of mesenchymal stromal cells on human umbilical vein endothelial cells in *in vitro *sepsis models

**DOI:** 10.1186/cc14075

**Published:** 2014-12-03

**Authors:** K Lund, J Peltzer, F Montespan, N Oru, E Vicaut, J Duranteau, J-J Lataillade

**Affiliations:** 1 Unité de Thérapie Cellulaire et Réparation Tissulaire, Institut de Recherche Biomédicale des Armées/Centre de Transfusion Sanguine des Armées, Clamart, France; 2 Laboratoire d'étude de la microcirculation, Université Paris - Diderot, Paris, France; 3 Service d'Anesthésie-Réanimation Chirurgicale, Centre Hospitalier Universitaire de Bicêtre, Le Kremlin-Bicêtre, France

## Introduction

Septic shock is a medical emergency that, despite the medical advances that have been made, still remains a major cause of hospital deaths. Cell therapy is an innovative field of research that could provide a therapy for sepsis. Mesenchymal stromal cells (MSC) are promising in cell therapy and more importantly for sepsis because of their immunosuppressive capabilities [1]. MSC have been shown by several groups to have a positive effect against sepsis *in vivo *[2-4]. It has been predicted that the MSC interact with macrophage to release IL-10 that in turns reduces inflammation [4]. Other groups have focused on the use of stimulated MSC to ameliorate their immunosuppressive capabilities [5]. The main stimulation of MSC has been the use of inflammatory stimulants like IFNγ. Our work focuses on the identification of effective MSC donors, whether primed with IFNγ or naïve, and the development of *in vitro *models that will predict how an MSC donor will act *in vivo*. We also want to eliminate the use of cells completely and use their secreted microvesicles as a therapy. The hypothesis is that the *in vitro *models will eliminate a noneffective MSC donor and allow us to identify the MSC donor that will have the greatest effect.

## Methods

We developed two *in vitro *models that are similar to what happens *in vivo *with WBC as they circulate in a septic patient. The first test is the adherence of WBC to a layer of HUVECs in the presence of MSC or microvesicles. The second is a permeability test to determine MSC ability to block the permeability of a HUVEC layer.

## Results

Our preliminary results have shown that we are able to identify, using our two *in vitro *models, which MSC donor would be an effective MSC for cell therapy.

## Conclusion

MSC and their paracrine factors have to the potential to be an effective therapy for sepsis, but one needs to identify an effective donor before use in cell therapy.
